# MicroRNA-100 functions as a tumor suppressor in cervical cancer via downregulating the SATB1 expression and regulating AKT/mTOR signaling pathway and epithelial-to-mesenchymal transition

**DOI:** 10.3892/ol.2021.13002

**Published:** 2021-08-17

**Authors:** Cuiping Huang, Xiaobo Qin, Na Zhao, Huijing Jin, Shuangjun Zhang, Haiyan Yang

Oncol Lett 20: 1336-1344, 2020; DOI: 10.3892/ol.2020.11686

Subsequently to the publication of the above article, an interested reader drew to the authors’ attention that a section of the data shown in Fig. 1C (Transwell assay data for HeLa cells), the “miR-100 inhibitor” panel appeared to be overlapping with the “miR-100 inhibitor” panel featured in Fig. 1D (showing the Transwell assay data for Ca-Ski cells).

The authors have checked their original data, and realized that the incorrect data were inadvertently selected for the “miR-100 inhibitor” panel in Fig. 1D. A corrected version of Fig. 1, including the correct data for Fig. 1D, is shown below. The authors are grateful to the Editor of *Oncology Letters* for granting them the opportunity to publish this corrigendum, and regret any inconvenience caused to the readership of the Journal.

## Figures and Tables

**Figure 3.**
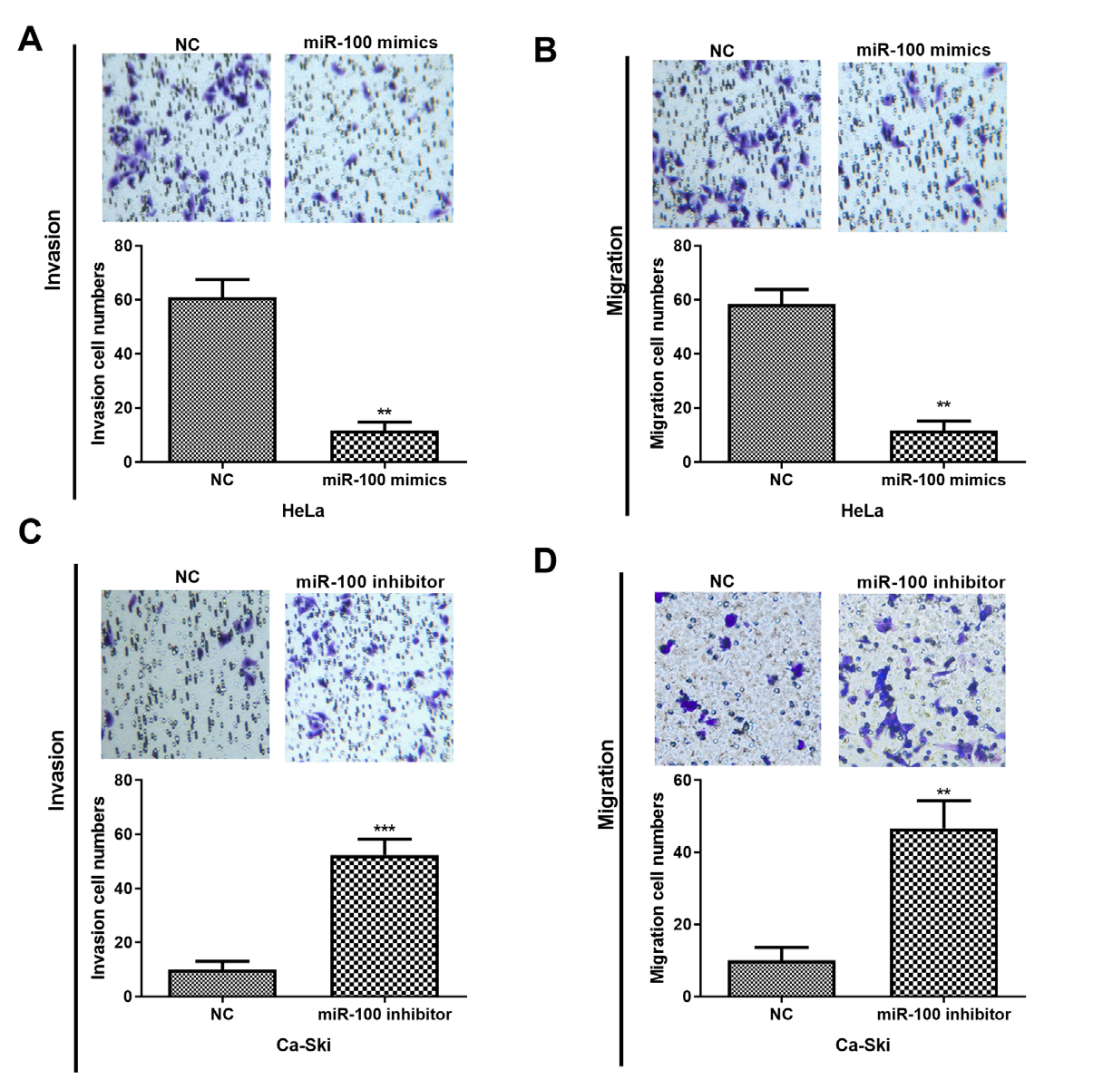
miR-100 restoration significantly represses CC cell invasion and migration. (A and B) The invasion and migration abilities of HeLa cells treated with miR-100 mimics were assessed by Transwell assays. (C and D) Transwell assay was performed to determine the invasion and migration capacities of Ca-Ski cells treated with miR-100 inhibitor. **P<0.01 and ***P<0.001. CC, cervical cancer.

